# Consensus Report on *Shigella* Controlled Human Infection Model: Clinical Endpoints

**DOI:** 10.1093/cid/ciz891

**Published:** 2019-12-09

**Authors:** Calman A MacLennan, Mark S Riddle, Wilbur H Chen, Kawsar R Talaat, Varsha Jain, A Louis Bourgeois, Robert Frenck, Karen Kotloff, Chad K Porter

**Affiliations:** 1 Bill & Melinda Gates Foundation, London, United Kingdom; 2 F. Edward Hébert School of Medicine, Uniformed Services University, Bethesda; 3 Center for Vaccine Development, University of Maryland School of Medicine, Baltimore, Maryland; 4 Center for Immunization Research, Department of International Health, Johns Hopkins Bloomberg School of Public Health, Baltimore, Maryland; 5 Bill & Melinda Gates Foundation, Seattle, Washington; 6 Enteric Vaccine Initiative, PATH, Washington, District of Columbia; 7 Division of Infectious Diseases, Cincinnati Children's Hospital Medical Center, Ohio; 8 Division of Infectious Disease and Tropical Pediatrics, Center for Vaccine Development, University of Maryland School of Medicine, Baltimore; 9 Enteric Disease Department, Naval Medical Research Center, Silver Spring, Maryland

**Keywords:** *Shigella*, controlled human infection model, human infection studies, endpoints

## Abstract

The *Shigella* controlled human infection model (CHIM) is valuable for assessing candidate *Shigella* vaccine efficacy and potentially accelerating regulatory approval. The *Shigella* CHIM is currently being conducted at 3 sites in the United States using *Shigella flexneri* 2a strain 2457T and *Shigella sonnei* strain 53G. Shigellosis can present variably as watery diarrhea alone or with dysentery, and can be accompanied by manifestations including fever, abdominal cramps, tenesmus, and malaise. For comparability, it is important to harmonize the primary clinical endpoint. An expert working group was convened on 2 February 2018 to review clinical data from *Shigella* CHIM studies performed to date and to develop a consensus primary endpoint. The consensus endpoint enabled “shigellosis” to present as severe diarrhea or moderate diarrhea or dysentery. The latter 2 criteria are met when concurrent with fever of 38.0°C and/or vomiting, and/or a constitutional/enteric symptom graded at least as “moderate” severity. The use of a blinded independent committee to adjudicate the primary endpoint by subject was also regarded as important. As safety of volunteers in challenge studies is of paramount importance and treatment timing can affect primary outcomes, a standard for early antibiotic administration was established as follows: (1) when the primary endpoint is met; (2) if a fever of ≥39.0°C develops; or (3) if the study physician deems it appropriate. Otherwise, antibiotics are given at 120 hours postinfectious challenge. The working group agreed on objective and subjective symptoms to be solicited, and standardized methods for assessing subject-reported severity of symptoms.

On 2 February 2018, a working group of 9 clinicians and scientists convened at the University of Maryland with the objective of reaching consensus on a primary clinical endpoint for the *Shigella* controlled human infection model (CHIM). The decision to convene the working group was made following a larger workshop held at the Bill & Melinda Gates Foundation in Washington, District of Columbia, on 27 November 2017. There are currently 3 institutions in the United States where the *Shigella* CHIM has been established and is being used: Johns Hopkins University (JHU) [1–5] the University of Maryland (UMD) [6–16], and Cincinnati Children's Hospital Medical Center (CCHMC) [17]. Clinical investigators from all 3 sites (Dr Kawsar Talaat [JHU]; Dr Karen Kotloff and Dr Wilbur Chen [UMD]; Dr Robert Frenck [CCHMC]) participated in the working group, together with others who have collectively been involved in conducting *Shigella* CHIM studies over the preceding 30 years (Dr Chad Porter, Dr Mark Riddle, and Dr Louis Bourgeois).

To date, the primary clinical endpoint in *Shigella* CHIM studies has varied across institutions, investigators, studies, and sponsors, ranging from mild to more severe disease [18]. In the ideal setting, the *Shigella* CHIM would be utilized to assist with up- and down-selection of a growing number of candidate vaccines against *Shigella* currently in development. However, to ensure a consistent and reproducible assessment of these candidates across multiple trials and trial sites, harmonization of clinical endpoints is necessary. In addition to the use of the *Shigella* CHIM as an early tool to screen multiple vaccine candidates, a precedent has been set for their potential use in supporting product licensure. For example, efficacy data from a typhoid CHIM study in Oxford, UK [19] contributed to a recent World Health Organization (WHO) Strategic Advisory Group of Experts on Immunization recommendation and subsequent prequalification [20] of the Typbar typhoid conjugate vaccine (Bharat Biotech). Similarly, data from a CHIM study were used to support licensure of an oral cholera vaccine for travelers [21]. Given the potential ability of the *Shigella* CHIM to support vaccine licensure, standardization of the primary endpoint is needed, as is the ability to execute multicenter studies in support of licensure.

The overriding concern when conducting a CHIM is to ensure the safety of study participants. Therefore, participation is restricted to healthy adult volunteers between 18 and 50 years of age. Second, the CHIM must be able to demonstrate the potential efficacy of a vaccine, which could be extrapolated to efficacy outcomes in field trials in an at-risk target population. Historically, the *Shigella* CHIM has been designed to provide treatment of all subjects regardless of detectable fecal shedding, to prevent spread of shigellosis to the community following discharge from the inpatient facility. The timing of treatment is generally 5 days for *S. sonnei* and *S. flexneri* 2a, to allow sufficient time for disease expression after the typical incubation period of 72 hours. Consideration might be given to treating later for *Shigella dysenteriae* considering its longer incubation period. Protocol-defined criteria are provided for early treatment of subjects who develop more severe disease, while ensuring subjects can meet the primary clinical endpoint. Establishing criteria for early treatment is an important component of model standardization. Furthermore, the infectious dose must be sufficient to allow the primary endpoint to be reached reproducibly in a predetermined proportion of naive unvaccinated volunteers, but not overwhelming to mask vaccine efficacy. While lacking objective supporting data, a primary endpoint attack rate of 60%–70% has traditionally been targeted to balance vaccine efficacy and practicable study design and execution. Furthermore, it has been observed that the field efficacy of a given vaccine is likely to exceed efficacy seen in a CHIM [15, 22–24]. Thus, a reasonable approach might be to use a sample size required to demonstrate vaccine efficacy with lower limit of 90% confidence interval >0 in relation to the primary clinical endpoint.

## GROUP REVIEW OF CLINICAL ENDPOINTS

A recent WHO expert consultation on preferred product characteristics for a *Shigella* vaccine concluded that such vaccines should prevent moderate to severe diarrhea, dysentery, and morbidity among children from 6 months to 5 years of age. Similarly, a travelers' diarrhea vaccine target product profile includes the prevention of moderate to severe diarrhea and dysentery in individuals traveling to moderate to high shigellosis risk regions of the world. These endpoints of moderate or severe disease in the target population were applied to the setting of the CHIM.

Workshop participants first reviewed and compared previously used clinical endpoints. Clinical data from individual *Shigella* CHIM study participants, including maximum frequency and volume of loose stools (grade 3–5 in a 1–5 grading system) ([25] and Talaat et al in this supplement) in any 24-hour period, total number and volume of loose stools, timing of loose stool episodes, presence of blood in stools, oral temperature, and constitutional/enteric symptoms were then reviewed by the group. Data were reviewed from a recent CHIM study to assess efficacy of a GSK/LimmaTech *S*. *flexneri* 2a bioconjugate vaccine [3], the evaluation of the *S*. *sonnei* CHIM at CCHMC [17], and historical *S*. *flexneri* 2a studies at UMD [8]. The group discussed the learning points from the presented findings and the relative merits of the different clinical endpoints currently used. A post hoc analysis of sets of CHIM study data analyzed using different endpoint criteria was presented, including a severity scoring system that can serve as an alternative to a traditional dichotomous endpoint [18].

## RECOMMENDATION FOR PRIMARY CLINICAL ENDPOINT

The working group reached a consensus recommendation for a primary clinical endpoint for future *Shigella* CHIM studies with *S. flexneri* 2a and *S. sonnei* (Table 1). In particular, the working group felt that the primary endpoint for CHIM studies assessing disease prevention should be focused on the spectrum of illness that corresponded with moderate to severe disease. With this perspective, participants concluded that 1 of 3 possible clinical scenarios should be required. The first, “severe diarrhea,” is a stand-alone endpoint determined solely by the number (≥6) or volume (>800 g) of loose or liquid stools produced in any rolling 24-hour period. The second, “moderate diarrhea,” consists of 4–5 or 400–800 g of loose or liquid stools in a 24-hour period, and a third endpoint, “dysentery,” is defined as ≥2 loose or liquid stools with gross blood in 24 hours.

**Table 1.** Primary Endpoint for Shigellosis in *Shigella* Controlled Human Infection Model

Primary Endpoint (Shigellosis)	Definition
1. Severe diarrhea	≥6 loose stools^a^ in 24 h OR >800 g loose stools in 24 h
2. Moderate diarrhea	[4–5 loose stools in 24 h OR 400–800 g loose stools in 24 h] AND [oral temperature ≥38.0°C^b^ OR ≥1 moderate constitutional/ enteric symptom^c^ OR ≥2 episodes of vomiting in 24 h]
3. Dysentery	≥2 loose stools with gross blood (hemoccult positive) in 24 h AND [oral temperature ≥38.0°C OR ≥1 moderate constitutional/ enteric symptom OR ≥2 episodes of vomiting in 24 h]

Participant must fulfill any 1 of the 3 possible endpoint scenarios to qualify as having reached the primary endpoint of shigellosis.

^a^Grade 3 to 5.

^b^Confirmed by 2 separate readings at least 5 minutes apart.

^c^See Table 2.

Both moderate diarrhea and dysentery require an additional clinical feature (common to both clinical scenarios) to qualify as the primary endpoint of “shigellosis”: a fever of at least 38.0°C, or either a constitutional or enteric symptom of moderate grade (defined as interfering with routine daily activities; Table 2) or ≥2 episodes of vomiting in 24 hours. A further qualifying symptom in these 2 scenarios was required so as not to include participants who met the stool criteria for moderate diarrhea or dysentery but who were otherwise systemically well with no impact on daily activities. In the cases reviewed, these endpoints differentiated the clinical illness from a milder presentation, which is not the focus of current vaccine development efforts, and enabled the models to be applied safely without undue risk to subjects.

**Table 2.** Constitutional/Enteric Symptoms That Qualify a Primary Endpoint of Moderate Diarrhea and Dysentery as Fulfilling the Definition of Shigellosis in *Shigella* Controlled Human Infection Model

Constitutional/Enteric Symptom
Nausea
Abdominal pain/cramping
Myalgia/arthralgia
Malaise

Symptom must be “moderate” in character, causing interference with routine activities (where “severe” symptoms are an inability to perform routine daily activities). Not included: anorexia, rigors/chills, tenesmus/fecal urgency, gas/flatulence, headache.

The group prioritized which constitutional and enteric symptoms to include as components of the “moderate diarrhea” and “dysentery” primary endpoint options, and which to exclude. It was agreed to include 2 “enteric” symptoms (nausea and abdominal pain/cramping) and 2 “constitutional” symptoms (myalgia/arthralgia and malaise). In the group's experience, these symptoms were the most disabling. Other enteric and constitutional symptoms were excluded because they were considered nonspecific, difficult to solicit accurately, and/or of mild severity. These included anorexia, headache, rigors/chills, tenesmus/fecal urgency, and gas/flatulence (Table 2). Nevertheless, the importance of collecting data on a full range of clinical parameters for secondary and post hoc analyses was emphasized by the group and there was agreement upon standardization of a core set of symptoms to include arthralgia, nausea, myalgia, headache, anorexia, abdominal cramps, and pain [18]. Fever, in the absence of gastrointestinal symptoms, was not considered a primary endpoint. In such instances, a search for other possible causes of fever should be conducted and full data from investigations reviewed by the study adjudication board, outlined below.

The group deliberated upon the relative value and challenges of including patient-reported symptoms as part of the clinical endpoint. It is recognized that the subjective nature of patient-reported outcomes can introduce study-to-study heterogeneity. However, their applicability and use in a clinical trial endpoint is of recognized value [26]. Important to the deliberation for the *Shigella* CHIM and studies of travelers' diarrhea is that such patient-reported symptoms are necessary components of a complex disease state that have clinically relevant meaning, driving care-seeking and functional disability. These symptoms and their subsequent functional sequelae can be impacted by vaccination. While more work is needed to validate outcomes that combine both objective measures and subjective symptoms, there was also a recognized need to collectively standardize the patient-reported symptoms of participants enrolled in CHIM studies, including the type and way in which they are collected and recorded by investigators. Further workshops and consensus meetings will be needed to assess these in the future.

## STOOL CULTURE

The group discussed the role of *Shigella* culture from the stool in relation to the subject meeting the primary endpoint. Although viewed as informative, there was consensus that a positive culture for *Shigella* was insufficient to determine shigellosis. There are clear historical examples of subjects shedding the challenge organism for multiple days with no *Shigella*-attributable signs or symptoms. Additionally, subjects may develop the signs and symptoms of shigellosis with no *Shigella* isolated from the stool. In these instances, it may be that early antibiotic initiation precludes the ability to culture the bacteria. However, while not a component of the endpoint criteria, quantitative culture or PCR of the challenge strain may be considered a relevant secondary outcome to evaluate vaccine performance.

## INOCULATION

Preparation of the challenge inoculum is beyond the scope of this review and may vary by strain and by lot. However, based on a reanalysis of the most recently completed studies with *S*. *flexneri* 2a strain 2457T [3] and *S. sonnei* strain 53G [17], results indicate that a minimum challenge dose of 1500 colony-forming units is required to ensure an attack rate of 60%–70% in naive volunteers using the shigellosis primary endpoint described above. To neutralize gastric acid, the challenge inoculum is given with a buffer solution consisting of 2 g sodium bicarbonate dissolved in 150 mL of sterile nonbacteriostatic water. Subjects receive 120 mL of bicarbonate buffer solution followed by the inoculum, which has been placed in 30 mL of the bicarbonate buffer solution. Volunteers fast for 90 minutes before and after inoculation.

## SELECTION OF VOLUNTEERS AND CONDUCT OF THE CHALLENGE

Appropriate screening of subjects is critical in human challenge studies. Investigators should ensure, to the best of their ability, that subjects are healthy, meet all eligibility criteria, and clearly understand the purpose and risks inherent in participating in a CHIM study. The study should be conducted in an inpatient setting to allow for careful observation and care of the subjects and to prevent transmission of the challenge strain to household contacts.

## ANTIBIOTIC TREATMENT

The current standard for antibiotic treatment is ciprofloxacin (500 mg by mouth twice daily for 3 days) starting 120 hours (ie, 5 days) after *Shigella* inoculation. There was consensus that early antibiotic intervention should be instituted as soon as a subject meets the primary endpoint (eg, any of the composite clinical outcomes). Additionally, it was thought that if a person developed a fever ≥39.0°C, early treatment is warranted. A final criterion for early antibiotic treatment was based on the principal investigator's clinical discretion (Table 3). There are occasional circumstances in which the a priori early treatment criteria are not met, but that for subject safety or because of unforeseen circumstances, subjects need to be treated early.

**Table 3.** Criteria for Early Treatment With Antibiotics in *Shigella* Controlled Human Infection Model

Criteria for Early Treatment
1. Meets primary endpoint definition of shigellosis
2. Temperature ≥39.0°C
3. Any other reason warranting early treatment in the physician's opinion

Treatment instigated as soon as any 1 criterion is fulfilled; otherwise, treat with antibiotics at 120 hours following ingestion of *Shigella* challenge dose.

Review of individual subject–level data from prior CHIM studies indicated that high fever following a *Shigella* infectious challenge normally heralds the onset of gastrointestinal symptoms, if not already present. The review also clarified that a primary endpoint may be met after initiation of antibiotics, and that the signs and symptoms of shigellosis and the assessment of the primary endpoint should continue beyond antibiotic initiation. A caveat to this is that if enteric/constitutional signs or symptoms do not commence until 24 hours or more after initiating antibiotics, these symptoms are likely not attributable to the *Shigella* infection; rather, they are more likely associated with acute postantibiotic diarrheal illness.

## ADJUDICATION BOARD

When the study involves groupwise comparisons, such as an efficacy trial comparing vaccinated and unvaccinated subjects, an adjudication board will review clinical data from all participants after completion of the inpatient period. The purpose of this board is to adjudicate, in blinded manner, the primary endpoint in individual *Shigella* CHIM studies. Members review de-identified patient-level data pertaining to the clinical signs and symptoms associated with shigellosis and document results. Additionally, the adjudication board may provide guidance on secondary and other endpoint classifications and/or review any protocol-specified entry criteria, adherence, and compliance issues to ascertain classification in the per-protocol and any post hoc study groupings. Voting members should be experienced in the execution of clinical trials and/or CHIM studies, independent from the sponsor and study-team, and should include clinicians.

## CONCLUSIONS

In conclusion, the recommended primary clinical endpoint for the *Shigella* CHIM has been determined by a group of experts based on the best available data from *Shigella* CHIM studies to date. Its ultimate validation will depend on results from future CHIM studies and may require adjustment in due course. However, it is thought that this outcome, perhaps used in conjunction with others, such as the recently proposed severity score [18], will differentiate disease in treatment groups and enable down-selection decisions for prototype vaccines and, potentially, support licensure of leading vaccine candidates.

## Notes

***Disclaimer.*** The views expressed in this article do not necessarily reflect the official policy or position of the Uniformed Services University, Department of the Navy, Department of Defense, nor the US government. This is a partial US government work. There are no restrictions on its use. Two of the authors (M. S. R. and C. K. P.) are employees of the US government. This work was prepared as part of official duties. Title 17 U.S.C. §105 provides that “[c]opyright protection under this title is not available for any work of the United States government.” Title 17 U.S.C. §101 defines a US government work as a work prepared by a military service member or employee of the US government as part of that person's official duties.

***Supplement sponsorship.*** This supplement is sponsored by the Bill & Melinda Gates Foundation.

***Potential conflicts of interest.*** C. K. P. reports a collaborative research development agreement with LimmaTech Biologics, outside the submitted work. K. R. T. has received grants for a *Shigella* vaccine and challenge study from LimmaTech Biologics, outside the submitted work. W. H. C. has received grants from Bill & Melinda Gates Foundation and Institut Pasteur, outside the submitted work. All other authors report no potential conflicts of interest. All authors have submitted the ICMJE Form for Disclosure of Potential Conflicts of Interest. Conflicts that the editors consider relevant to the content of the manuscript have been disclosed.
